# Postural adjustments to self-triggered perturbations under conditions of changes in body orientation

**DOI:** 10.1007/s00221-023-06671-0

**Published:** 2023-07-21

**Authors:** Francesco Pascucci, Paola Cesari, Matteo Bertucco, Mark L. Latash

**Affiliations:** 1 Department of Neurosciences, Biomedicine and Movement Sciences, University of Verona, Via Felice Casorati 43, 37131 Verona, Italy; 2 Department of Engineering for Innovation Medicine, University of Verona, Verona, Italy; 3 Department of Kinesiology, The Pennsylvania State University, University Park, PA 16802 USA

**Keywords:** Postural control, Referent coordinate, Anticipatory postural adjustments, Compensatory postural adjustments, Reciprocal activation, Coactivation

## Abstract

We studied anticipatory and compensatory postural adjustments (APAs and CPAs) associated with self-triggered postural perturbations in conditions with changes in the initial body orientation. In particular, we were testing hypotheses on adjustments in the reciprocal and coactivation commands, role of proximal vs. distal muscles, and correlations between changes in indices of APAs and CPAs. Healthy young participants stood on a board with full support or reduced support area and held a standard load in the extended arms. They released the load in a self-paced manned with a standard small-amplitude arm movement. Electromyograms of 12 muscles were recorded and used to compute reciprocal and coactivation indices between three muscle pairs on both sides of the body. The subject’s body was oriented toward one of three targets: straight ahead, 60° to the left, and 60° to the right. Body orientation has stronger effects on proximal muscle pairs compared to distal muscles. It led to more consistent changes in the reciprocal command compared to the coactivation command. Indices of APAs and CPAs showed positive correlations across conditions. We conclude that the earlier suggested hierarchical relations between the reciprocal and coactivation command could be task-specific. Predominance of negative or positive correlations between APA and CPA indices could also be task-specific.

## Introduction

Human vertical posture is inherently unstable in the field of gravity because of the high center of mass, relatively small support area, and multiple joints in the lower limbs. Standing is additionally challenged by changes in external forces acting on the body. In this context, we imply under stability an ability of the body to avoid losing balance, i.e., keeping time-varying salient mechanical variables within specific ranges, under natural spontaneous changes in body states and in the presence of moderate changes in the external forces (reviewed in Latash and Zatsiorsky 2016). When such external perturbations are triggered by the person’s own action, two types of postural adjustments are seen, addressed as anticipatory postural adjustments (APAs) and compensatory postural adjustments (CPAs) (Belenkiy et al. 1967; Cordo and Nashner 1982; Massion 1992; Kaewmanee and Aruin 2022). APAs are initiated prior to the perturbation and reflect person’s prediction of the mechanical effects of the perturbation on vertical posture. CPAs are initiated a few tens of ms after the perturbation and reflect its actual mechanical effects on the body.

A number of studies explored changes in postural adjustments induced by manipulations of such parameters as the magnitude of the perturbation, the magnitude of action associated with the perturbation, and postural stability (Aruin and Latash 1995; reviewed in Kaewmanee and Aruin 2022; Bertucco and Cesari 2010; Bertucco et al. 2013). In this study, we explored the effects of additional factors that rarely get manipulated in studies of postural adjustments on APAs and CPAs, namely torso rotation and direction of a standard perturbation triggered by a standard action in conditions without changes in postural stability. This happens commonly in everyday life when interactions with external objects are not organized along the anterior–posterior or medio-lateral direction. Effects of torso rotation may also have clinical significance, in particular in persons with chronic low-back pain who may show increased pain level and associated limits of torso rotation (Taniguchi et al. 2017).

To achieve this goal, we used a load-release task from extended arms (cf. Aruin and Latash 1995; Aruin et al. 2001) and varied the initial rotation of the whole body around the vertical axis without moving the feet. This manipulation led to two major consequences. First, the change in the external moment of force associated with the load release had systematically varying components in a sagittal plane and in a frontal plane. Second, the initial body configuration changed with larger changes taking place in more proximal joints (e.g., the hip joints) compared to more distal joints (e.g., the ankle joints). With an increase in the initial body rotation, the first factor was expected to lead to smaller perturbations in a sagittal plane and larger perturbations in a frontal plane. The second factor was expected to have smaller effects on distal muscles and larger effects on proximal muscles, which naturally aligned their line of action with changes in the direction of the expected perturbation. Based on these considerations, we expected only the proximal muscles to show larger involvement during body rotation from its natural position in both APAs and CPAs (Hypothesis 1).

Rotation of the body in a particular direction, e.g., to the left, without moving the feet leads to larger motion in more proximal joints on the opposite side of the body (e.g., right). Even though the muscles implied include the ones from the lower extremity to the cranio-cervical region, the muscles opposite to the body rotation direction and located at the level of the trunk are the one massively involved in the action (Neumann 2010). Hence, we expected larger adjustments to body rotation in both APAs and CPAs seen in those muscles as compared to muscles on the other, ipsi-rotational, *Body Side* (Hypothesis 2).

Overall, APAs are commonly compensating for only a fraction of the mechanical effects of an expected perturbation on the body, such that the effects of the perturbation are attenuated but never fully canceled out (Massion 1992). This strategy may be a reflection of the fact that, in certain conditions, APAs themselves may turn into perturbing factors, in particular during standing on a narrow support or otherwise challenging conditions (Slijper et al. 2002; Krishnamoorthy and Latash 2005). Besides, smaller APAs allow predicting the direction of the residual perturbation and, therefore, make it possible to pre-program CPAs with confidence.

A number of recent studies have shown negative correlation between the indices of magnitude of APAs and CPAs (Krishnan and Aruin 2011; Kaewmanee et al. 2020). These results are logical consequences of the assumed functions of the two adjustments: larger APAs are more effective in dealing with the anticipated perturbation and its residual mechanical effects on the body are reduced and require smaller CPAs. We expected that effects of changes in the initial body position would also conform to this rule, and parameters of APAs and CPAs would show negatively co-varying changes, i.e., an increase in APAs with a change in the torso rotation would be accompanied by a decrease in CPAs (Hypothesis 3).

**Fig. 1:**
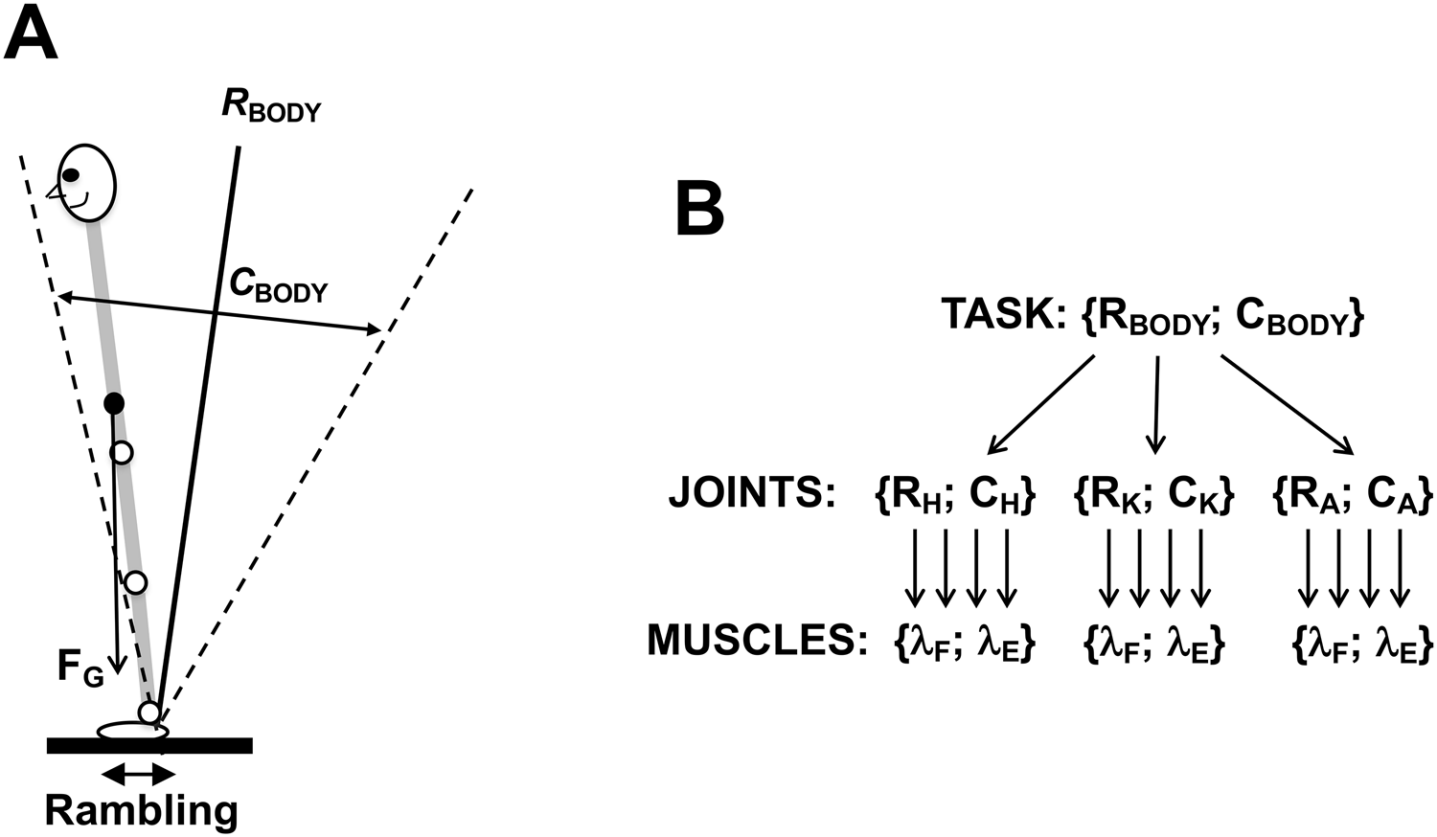
**A** A schematic illustration of the control of vertical posture in a sagittal plane. Posture is controlled with respect to a time-varying spatial coordinate (rambling). Referent orientation of the body with respect to that coordinate is defined by the reciprocal command (*R*-command). The difference between the actual and referent body orientations produces moment of force balancing that of the gravity force. This transformation is mediated by the coactivation command (*C*-command). **B** The *R*- and *C*-commands at the task (body) level are mapped on {R; C} commands for the joints (*H* hip, *K* knee, *A* ankle), which project of control variables for the muscles (*λ*) for the flexor (F) and extensor (E) muscles. Note that all these transformations are abundant with more variables at lower levels compared to those at higher levels

To quantify APAs and CPAs, we used variables reflecting changes in activation of agonist–antagonist muscle pairs, namely changes in reciprocal activation and coactivation indices (*R*-index and *C*-index, cf. Piscitelli et al. 2017; Nardini et al. 2019; Cesari et al. 2022). These two indices reflect two basic control variables within the equilibrium-point hypothesis (Feldman 1966, 1986) and its development as the idea of control with spatial referent coordinates for the involved effectors (reviewed in Latash 2010; Feldman 2015). Figure 1 illustrates the control of posture using a simplified example of body motion in the anterior–posterior direction only (a similar illustration can be drawn for other directions, e.g., for the medio-lateral direction). Body balance is organized with respect to a moving coordinate as shown with the double arrow under the drawing in panel A of Fig. 1. Migration of this point was defined is a classical study by Zatsiorsky and Duarte (2000) under the name of *rambling*. This coordinate can be viewed as a linear referent coordinate of the body (RC). Balancing about this RC involves defining referent orientation of the body (shown as the solid slanted line) with one of the two basic commands, the reciprocal command (*R*-command). The difference between the referent and actual body orientations translates into moment of force depending on the other basic command–coactivation command (*C*-command). This scheme involves both linear and rotational components of the RC vector, which translate into pairs of *R*- and *C*-commands to the individual joints and further—to muscles (as shown in panel B of Fig. 1). Note that all the control variables shown in Fig. 1 are time-varying ensuring postural stability about time-varying coordinates in space. Recent studies have suggested the importance of both corticospinal and vestibulospinal pathways for defining RC values during postural and locomotion tasks (Zhang et al. 2018; Shoja et al. 2023).

Earlier studies have shown that APA adjustments are associated primarily with adjustments of the *C*-index in muscles crossing the ankle joint (Slijper and Latash 2000, 2004; Piscitelli et al. 2017). Hence, we expected the *C*-index to be more sensitive to variations in the perturbation direction (Hypothesis 4).

In addition, we explored effects of postural instability induced by standing on a narrow support on both APAs and CPAs. Earlier studies have shown reduced APAs to a standard perturbation in similar conditions (Nouillot et al. 1992; Aruin et al. 1998), suggesting that CPA could be increased to handle the increased residual postural perturbation. However, those studies did not manipulate direction of the perturbation. Therefore, this was an exploratory manipulation.

## Methods

### Participants

Twelve healthy young men of age 24.81 ± 2.77 years (means ± SD) without any known motor disorders or history of musculoskeletal injuries volunteered for this study. All participants had no history of neurological or musculoskeletal injury and had normal or corrected-to-normal vision. All participants were right-side dominant. The study protocol was performed in accordance with the Declaration of Helsinki and all participants gave their written informed consent. Ethical approval for the study was obtained from the Institutional Review Board at the Department of Neurosciences, Biomedicine and Movement Sciences of the University of Verona (n.26.R2/2021).

### Apparatus

The signals from a floor-embedded force platform (model OR-5, AMTI, USA: 90 × 90 cm) were amplified and used to measure reaction forces in three orthogonal directions: F_Z_ along the direction of gravity, F_X_ parallel to the ground along the frontal plane, and F_Y_ parallel to the ground along the sagittal plane. EMG signals were recorded using a wireless low-power signal conditioning electronics device (Zero Wire Aurion, Milan, Italy). Data were acquired using a 1000 Hz sample frequency for the analog-to-digital conversion. Pairs of EMG electrodes were attached over six muscles on both sides of the body: Tibialis Anterior (TA), Soleus (SO), Rectus Femoris (RF), Biceps Femoris (BF), Erector Spinae (ES), and Rectus abdominis (RA). This muscle selection (from possible agonists) was relatively arbitrary and defined by the limited number of channels in our recording system and a previous study (Bertucco et al. 2021). Electrodes were attached to the skin, after removing hairs and rubbing the skin with denatured alcohol to obtain a clean signal. Electrode placement followed recommendation by SENIAM project (Hermens et al. 2000).

Two wooden boards were used to produce the “Unstable” and “Stable” conditions. The boards were positioned on the force platform and their dimensions were 45 cm long and 45 cm wide. One board was in contact with the force platform by means of a narrow beam glued along the midline having 45 cm in length and 6 cm in width. The total height of the board with the narrow beam was 5 cm. The other one was a 5 cm-high solid block of wood. The first board was used to induce the “Unstable” condition, while the latter board was used for the “Stable” condition. For the “Unstable” condition, participants had the feet positioned perpendicular to the narrow beam provoking postural instability along the sagittal plane. A sphere made of aluminum, with a mass of 1.9 kg, was used as the load.

### Procedure

**Fig. 2:**
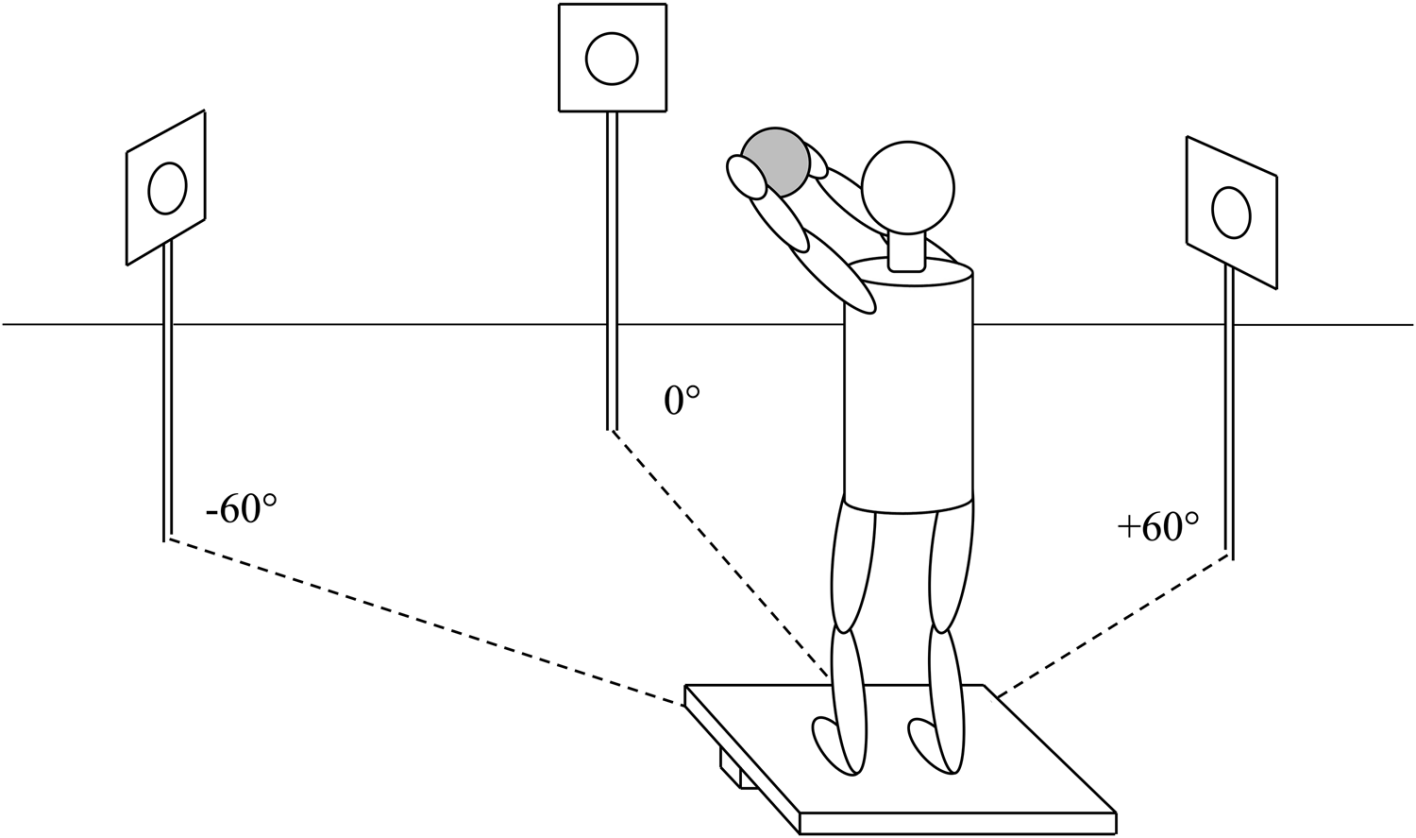
Schematic representation of the experimental setup. Participants were asked to stand comfortably on one of the two boards (the “Unstable” board is illustrated), with arms stretched forward parallel to the ground and fingers outstretched, holding the sphere between their palms. They were instructed to turn the body without moving their feet toward one of the three directions as shown in the figure and drop the sphere at their own rate by quickly abducting bilaterally both upper arms along the horizontal plane with a small-amplitude action

The initial position for the participants was to stand comfortably and quietly on one of the two boards, keeping arms stretched forward parallel to the ground and holding the sphere between the palms with fingers extended. The subjects looked at a 5 cm in diameter red dot placed 5 m away at eye level. There were three different positions for the red dot: one was located in front of the participants, defined as "0° direction” and the other two at an angle of 60° to the right (+ 60°) and 60° to the left (–60°) with respect to the central position (see Fig. 2). The subjects were instructed to keep the gaze straight and to align it with the red dot position using whole-body rotation. Compliance with this instruction was ensured by an experimenter watching the subjects.

In each trial, participants were instructed to turn the body toward one of the three directions without moving their feet that were kept in the same position marked on the platform. As soon as the instructed body position was achieved, a recorded voice notified the initiation of the trial. Participants were then allowed, in a self-paced manner, to drop the sphere by quickly abducting bilaterally both upper arms along the horizontal plane with a small-amplitude movement. After the sphere was released, participants were required to maintain the final posture with stretched open arms for 1 s until the recorded voice notified the end of the trial. The order of presentation for both platform stability (“Stable” and “Unstable”) and body orientation (0°, + 60° and –60°) was randomized across participants. Note that we use here the words *Stable* and *Unstable* as labels; across all conditions, the participants maintained stable initial posture and only trials without losing balance after the load release were accepted.

The task was performed six times for each direction, for each of the two stability conditions, for a total of 36 trials. We limited the number of trials per conditions to six to keep the duration of the experiment relatively short and avoid fatigue. Two-minute break was given every six trials to avoid fatigue. Trials where balance was lost in the “Unstable” condition were rejected and repeated. Before data collection, the natural foot position was marked on both boards with tape and maintained throughout the entire experiment. Two trials for each direction were given as practice trials.

### Data analysis

Data were processed offline in MATLAB software (R2021b, version 9.11). Force plate signals were filtered with a 10 Hz low-pass, tenth-order, zero-phase digital Butterworth filter. EMG signals were rectified and filtered with a 5–50 Hz band-pass, sixth-order, zero-phase digital Butterworth filter. For each trial, the time release of the load was calculated as the point in time when the derivative of force in the Z-direction (perpendicular to the ground) crossed 5% of its maximum value, prior to its peak. The instant of release was defined as time zero, t_0_. The EMG activity for each muscle was integrated using a trapezoidal numerical integration, with the function “trapz” in Matlab. Anticipatory Postural Adjustments (APA) were defined from –150 to + 50 ms with respect to t_0_, while Compensatory Postural Adjustments (CPA) were defined from + 50 to + 250 ms with respect to t_0_. Integrals, identified as ∫APA and ∫CPA, were further corrected by subtracting background activity. This value, addressed as *Baseline*, was calculated for each trial as the integral of the EMG signal in a 200-ms time window before the APA time window

$$\int \mathrm{APA}= {\int }_{-150}^{50}\mathrm{EMG} dt-\mathrm{Baseline}$$

$$\int \mathrm{CPA}= {\int }_{50}^{250}\mathrm{EMG }dt-\mathrm{Baseline}.$$

We subtracted the baseline values to explore changes in muscle activation during APAs and CPAs. The ∫APA and ∫CPA values for each muscle were normalized by the maximal absolute magnitude for each phase across all trials for each participant separately. As a result, all indices were kept within the range from –1 to + 1. Because of the unavoidable problems with any method of defining the exact timing of APAs and CPAs due to the spontaneous EMG variations, we elected to use fixed time intervals as those characteristics for the two adjustments to perturbation. The time intervals were selected based on earlier studies (Chen et al. 2018) as well as on pilot experiments. The *C* and *R* indexes were also computed, for APA and CPA separately, to address coactivation and reciprocal changes in the activity of the agonist–antagonist (ventral–dorsal) pairs acting at each joint. The three muscle pairs were TA–SO, RF–BF, and RA–ES. Specifically, the *R* and *C* indexes were quantified as (Piscitelli et al. 2017)

$$R=\int \mathrm{EM}{\mathrm{G }}_{\mathrm{V}}- \int \mathrm{EM}{\mathrm{G }}_{\mathrm{D}}$$

$$C\,\, = \left\{ {\begin{array}{*{20}l} 0 & {if{\text{ }}\int {EMG_{V} } \,and\,\int {\,EMG_{D} } \,have\,{\text{ }}different{\text{ }}signs} \\ {\min \left\{ {\left| {\int {EMG_{V} } } \right|;\,\left| {\int {EMG_{D} } } \right|} \right\}} & {if{\text{ }}\int {EMG_{V} } \,and\,\int {\,EMG_{D} } \,have\,{\text{ }}same{\text{ }}signs} \\ \end{array} } \right.$$

where V stands for Ventral and D stands for Dorsal. The *R* and *C* indexes for each pair of muscles were normalized by the absolute maximum magnitude for each phase across all experimental trials.

### Statistical analysis

Statistical analysis was performed using SPSS 16.0 (IBM Corp., Armonk, NY, USA) and RStudio (RStudio Inc., Version 1.4.1106, Boston, MA). Data in the text, Tables, and Figures, unless otherwise stated, are presented as means ± SD. Normality of the data was confirmed by inspection of density and Q–Q plots, Shapiro–Wilk test was performed, and data distribution (skewness and kurtosis) was assessed. When normality was violated, non-parametric statistical tests were performed (such as Wilcoxon’s signed-rank test).

To test Hypotheses 1, 2, and 4, ANOVA with repeated measures for the *C* and *R* indices and for APAs and CPAs was used with the factors: *Direction* (three levels: + 60°; 0°; –60°), *Muscle-Pair* (three levels: TA–SO, RF–BF, and RA–ES), and *Body Side* (two levels: right and left) for each level of instability (“Stable” and “Unstable”) separately. Pairwise contrasts with Bonferroni corrections were used to explore significant interactions. Critical p value was set at 0.05.

To test correlation between APAs and CPAs (cf. Hypothesis 3), regression analysis was performed for each subject separately. We report coefficients of determination *R*^2^, as well as results of the Wilcoxon’s signed-rank test.

## Results

**Fig. 3:**
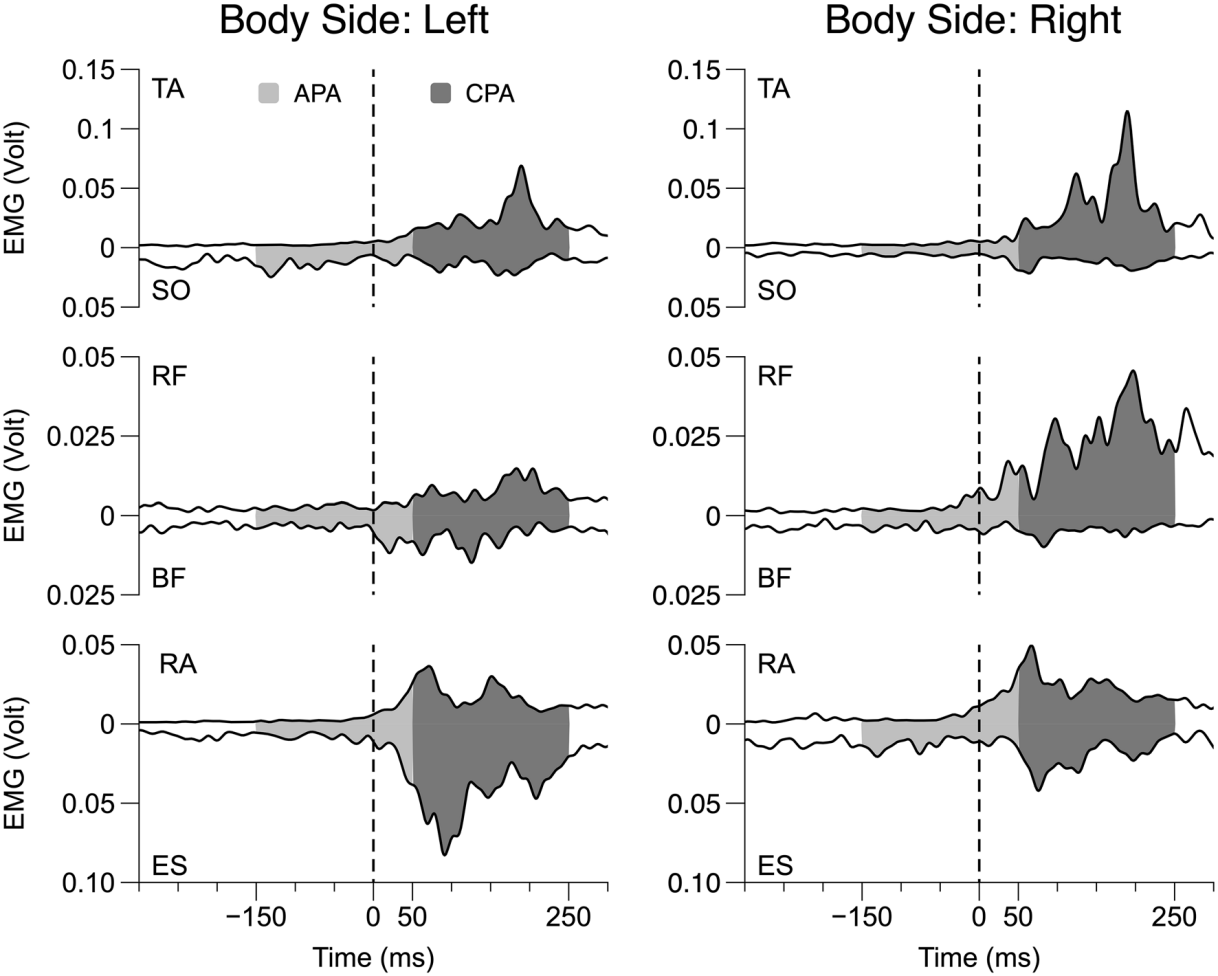
Filtered EMG traces (six trials averaged) for a representative participant in the -60° direction and Unstable condition for *Left and Right Body Side*. The vertical dashed line corresponds to the release of the load. EMG in Volt units. Anticipatory postural adjustments (APA) and compensatory postural adjustments (CPA) are highlighted in light and dark gray, respectively

**Fig. 4:**
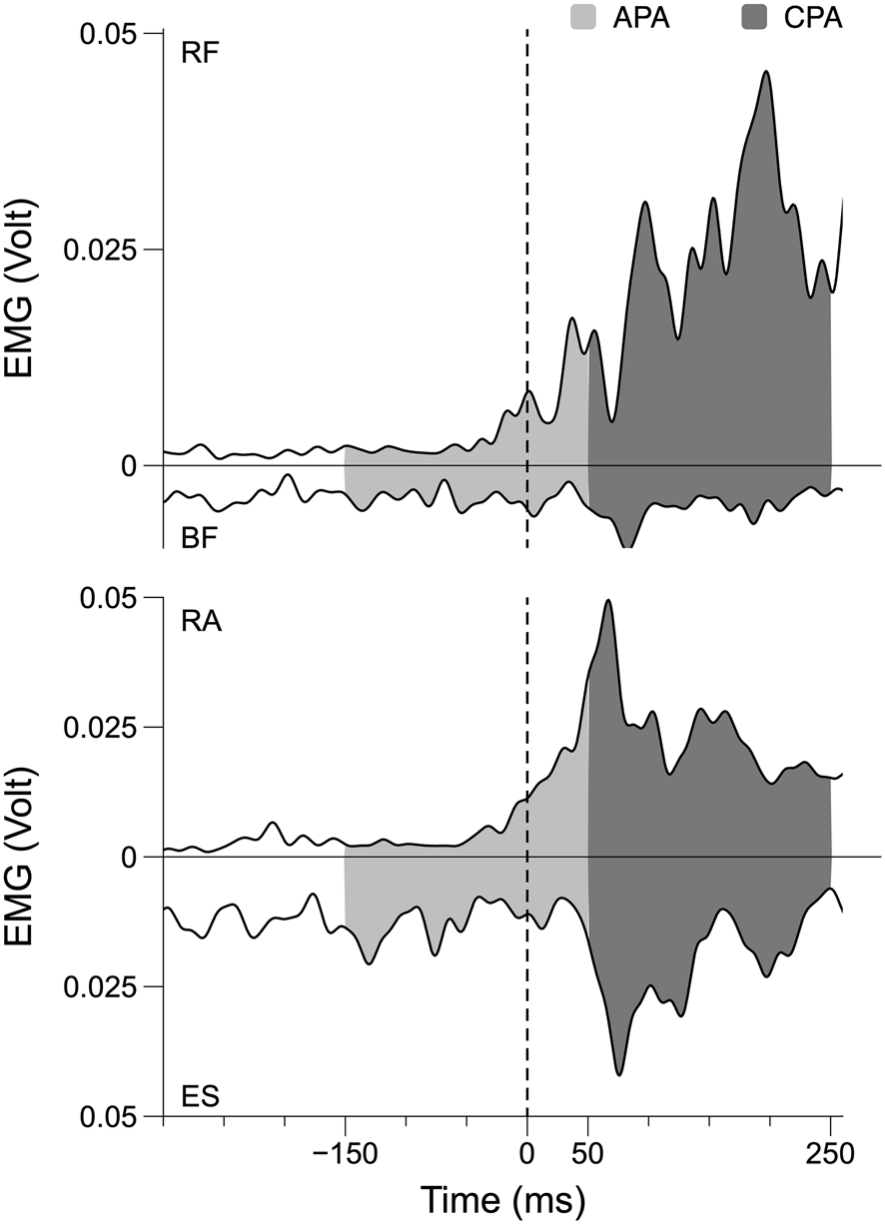
Filtered EMG traces (6 trials averaged) of RF, BF, RA, and ES for a better representation of the APA and CPA timing. The figure shows the traces for a representative participant in the -60° direction and Unstable condition for *Right Body Side*. The vertical dashed line corresponds to the release of the load. EMG in Volt units. Anticipatory postural adjustments (APA) and compensatory postural adjustments (CPA) are, respectively, highlighted in light and dark gray

Consistent EMG patterns in the leg and trunk muscles were seen across subjects timed to the moment of load release (t_0_) (Figs. 3 and 4).

These included an increase in the activation level of TA, RF, and RA prior to t_0_, typically accompanied by a drop in the baseline activation level of SO, BF, and ES (APA). Following the load release, there was typically an increase in the EMG of TA, SO, RF, and RA (CPA). Integrated indices for all six muscles during both APA and CPA on both sides of the body across conditions are presented in Table 1 (means ± SD across subjects). To test the specific hypotheses formulated in the Introduction, the EMG indices for individual muscles were converted into the *R*– and *C*–indices reflecting reciprocal activation and coactivation within agonist–antagonist muscle pairs (see Methods).

**Table 1:** Muscle activation indices associated with load release

Muscle/postural adjustment	APA	CPA
TA	0.059 ± 0.013	0.161 ± 0.014
SO	−0.013 ± 0.01	0.037 ± 0.013
RF	0.135 ± 0.014	0.206 ± 0.014
BF	−0.135 ± 0.012	−0.020 ± 0.018
RA	0.084 ± 0.018	0.257 ± 0.019
ES	−0.108 ± 0.013	−0.01 ± 0.023

Normalized integral values for the six muscles during both anticipatory postural adjustments (APA) and compensatory postural adjustments (CPA), averaged between the two sides of the body, across conditions and across subjects (mean ± standard deviation)

*TA* tibialis anterior, *SO* soleus, *RF* rectus femoris, *BF* biceps femoris, *RA* rectus abdominis, *ES* erector spinae

### Analysis of APAs

**Fig. 5:**
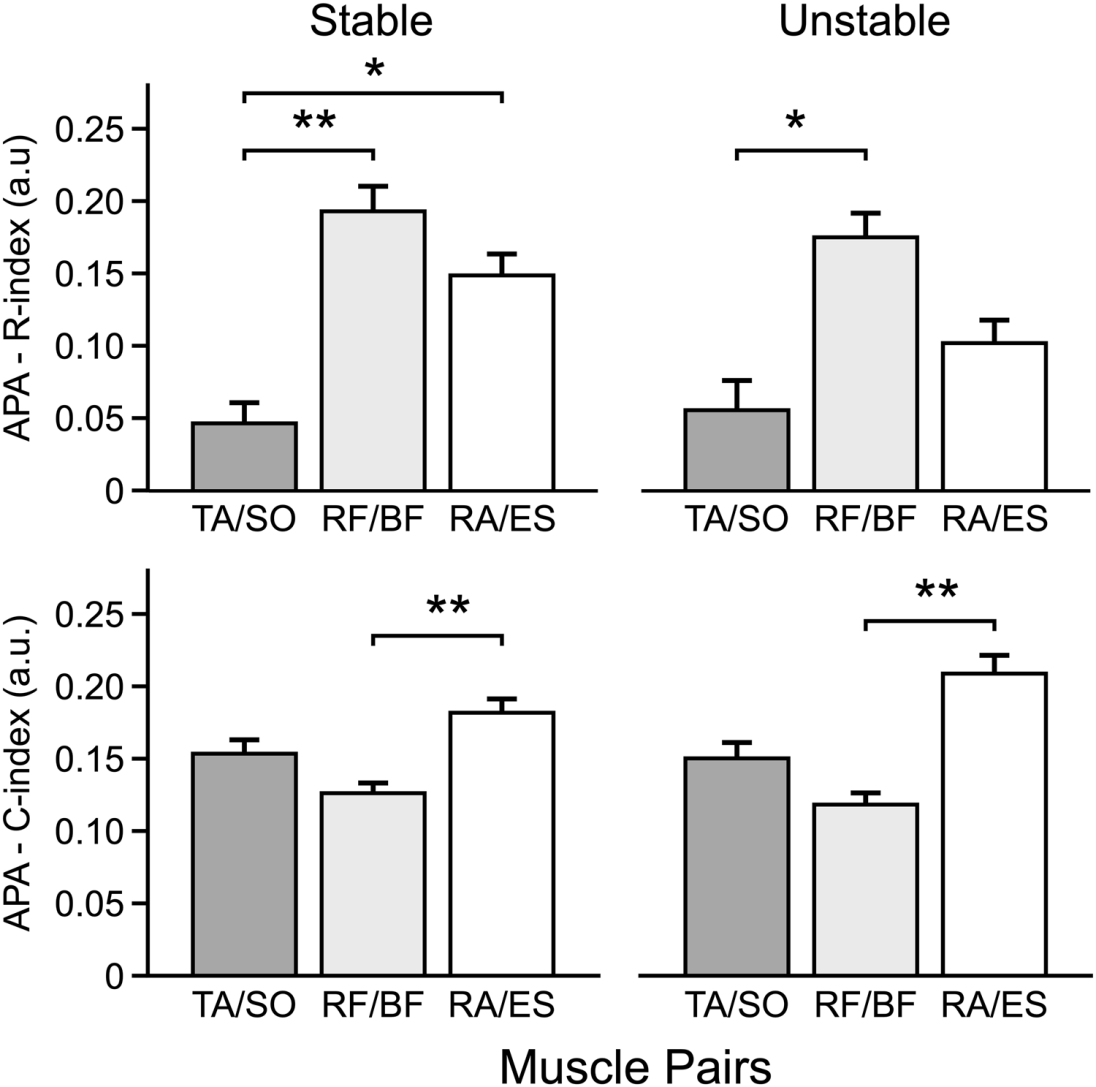
*R*-index (upper row) and *C*-index (bottom row) values related to anticipatory postural adjustments (APA) for the agonist–antagonist pairs acting at the ankle (TA/SO, dark gray), knee (RF/BF, light gray), and hip (RA/ES, white) joints for the Stable Condition (left column) and Unstable Condition (right column). Histograms depict the mean value and the standard error. *significant effects at *p* < 0.05, ** significant effects at *p* < 0.01. *TA* tibialis anterior; *SO* soleus, *RF* rectus femoris, *BF* biceps femoris, *RA* rectus abdominis, *ES* erector spinae

During the APAs, across the Stable and Unstable conditions, there were consistently higher indices of reciprocal inhibition (*R*-index) in the RF–BF muscle pair as compared to those in the TA–SO muscle pair. The *R*-index for the RA–ES pair was, on average, lower than in the RF–BF and higher than in TA–SO, but the difference was significant for the RF–BF vs. TA–SO comparison in the Stable condition only. These results supporting Hypothesis 1 are illustrated in the upper panels of Fig. 5. The results were confirmed by main effects of *Muscle-Pair* in the Stable condition [F_(2,22)_ = 9.72, *p* < 0.001] and in the Unstable condition [*F*_(2,22)_ = 5.79, *p* < 0.01]. Pairwise contrasts confirmed the mentioned differences between muscle pairs (*p* < 0.05).

**Fig. 6:**
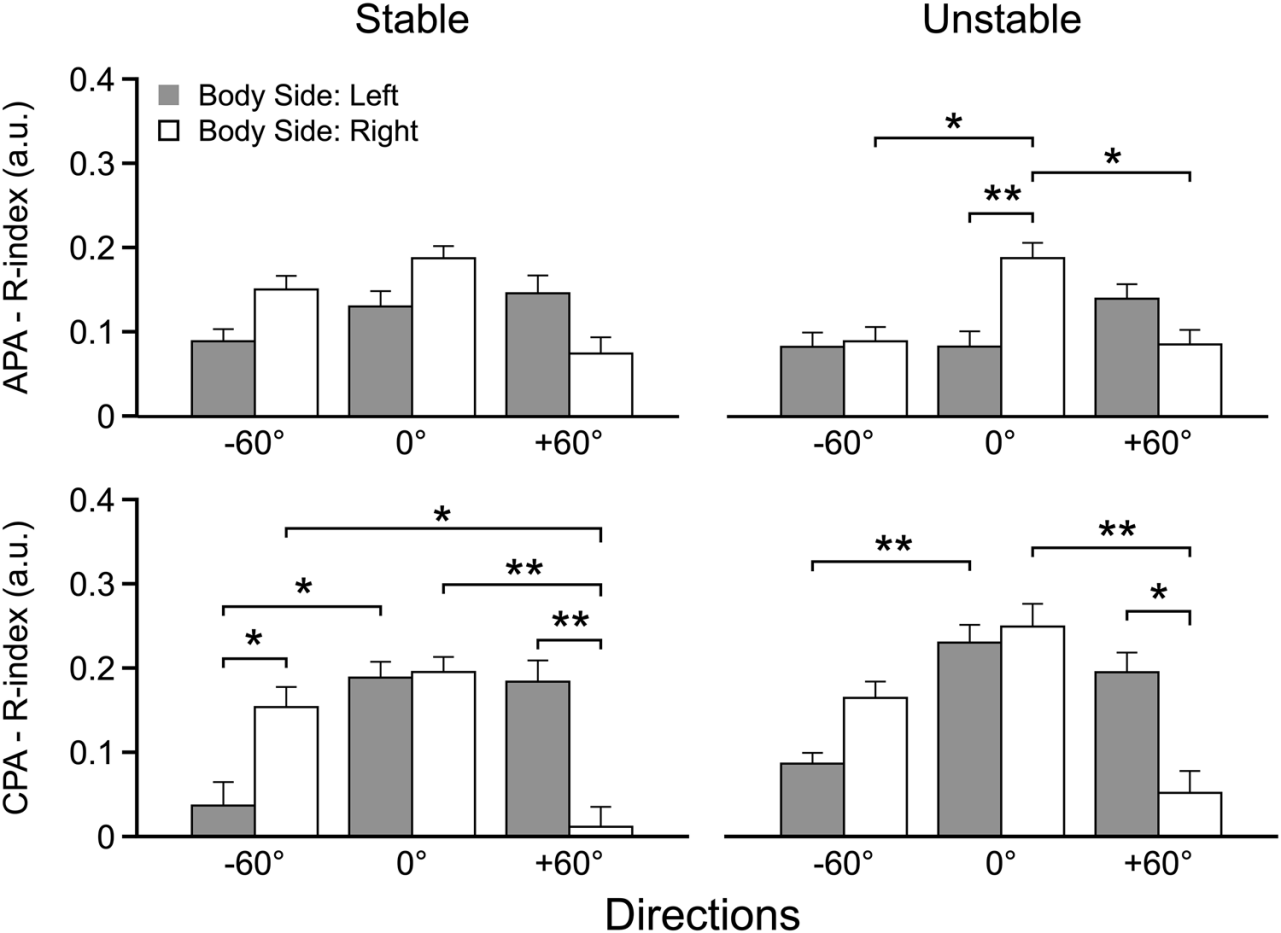
*R*-index values as a function of the release angle of the sphere (-60°, 0°, and 60°) for anticipatory postural adjustments (APA, upper row) and compensatory postural adjustments (CPA, bottom row) for the Stable Condition (left column) and Unstable Condition (right column). Gray and white plots represent left and right body sides, respectively. Histograms depict the mean value and the standard error. *significant effects at *p* < 0.05, **significant effects at *p* < 0.01

Moreover, the *R*-index showed effects of the *Body Side* and condition illustrated in the two graphs on top of Fig. 6. The effects showed similar patterns in the Stable and Unstable conditions but reached significance for the Unstable condition only. They included higher indices for the 0° body orientation on the right side of the body compared to the left side, and higher indices for the 0° orientation compared to both –60° and + 60° orientations. These effects were confirmed by significant interaction *Body Side* × *Direction* [*F*_(2,22)_ = 4.627 *p* < 0.025 for the Stable condition and, *F*_(2,22)_ = 8.22, *p* < 0.005 for the Unstable condition] with pairwise contrasts confirming the mentioned differences for the Unstable condition only.

The *C*-index showed a very different pattern with the lowest values for the RF–BF pair, which were significantly different from those in the RA–ES pair (effect of *Muscle-Pair*, *F*_(2,22)_ = 8.29, *p* < 0.005 for the Stable condition and *F*_(2,22)_ = 7.49, *p* < 0.005 for the Unstable condition) (the lower panels of Fig. 5). Differently than *R*-index, neither *Body Side* nor *Direction* indicated specific patterns, which fails to support Hypothesis 4.

### Analysis of CPA

**Fig. 7:**
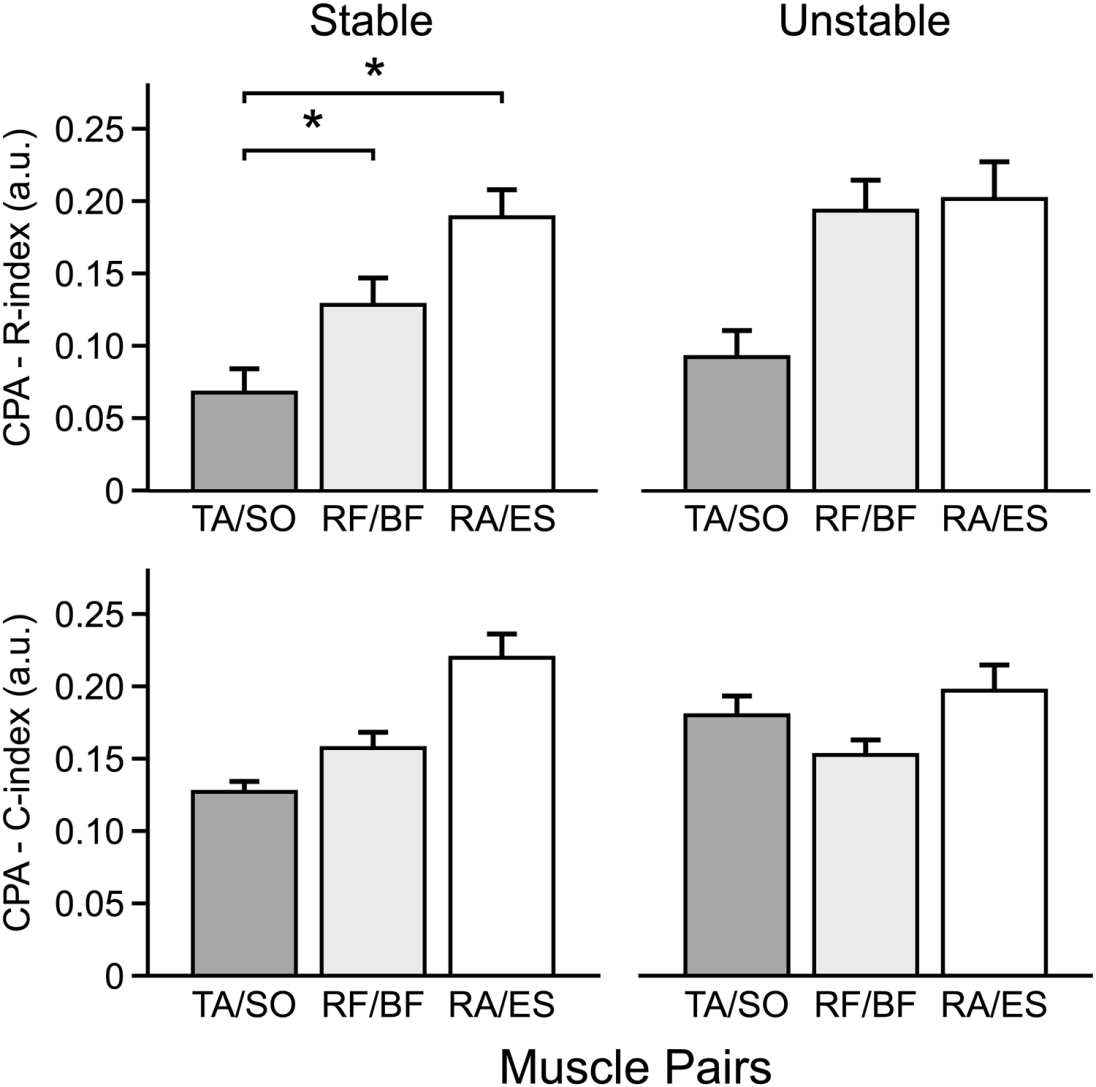
*R*-index (upper row) and *C*-index (bottom row) values related to compensatory postural adjustments (CPA) for the agonist–antagonist pairs acting at the ankle (TA/SO, dark gray), knee (RF/BF, light gray), and hip (RA/ES, white) joints for the Stable Condition (left column) and Unstable Condition (right column). Histograms depict the mean value and the standard error. *significant effects at *p* < 0.05, **significant effects at *p* < 0.01. *TA* tibialis anterior, *SO* soleus, *RF* rectus femoris, *BF* biceps femoris, *RA* rectus abdominis, *ES* erector spinae

During the CPAs, for *R*-index, the effects were similar across the Stable and Unstable conditions with more significant comparisons for the Stable condition. The higher magnitudes of the *R*-index were seen for the 0° body orientation (main effect of *Direction*, *F*_(2,22)_, = 6.52, *p* < 0.01 for Stable and *F*_(2,22)_, = 9.49, *p* < 0.001 for Unstable). When the body orientation differed from 0°, rotation to the left induced higher values of the *R*-index in the right-side muscles, whereas rotation to the right showed higher values for the left-side muscles [*Body Side* × *Direction* interaction, *F*_(2,22)_ = 9.84 *p* < 0.001 for Stable and *F*_(2,22)_ = 6.77 *p* < 0.01 for Unstable]. In other words, the values were significantly higher on the side of the body opposite to the rotation direction (cf. Hypothesis 2), and this effect was particularly pronounced for the Stable condition (the lower panels of Fig. 4). Moreover, as predicted by Hypothesis 1, the *R*-index for the RA–ES muscle pair was, on average, higher than in the RF–BF, and the latter higher than TA–SO muscle pair in the Stable condition. The same analysis for Unstable condition indicated a similar pattern but without reaching the significance. The results are illustrated in the upper panels of Fig. 7 and confirmed by the main effects of *Muscle-Pair* only in the Stable condition *F*_(2,22)_ = 7.92, *p* = 0.005 (Fig. 5).

During CPAs, for the *C*-index, no differences were detected between muscle pair in the Stable condition, even though the main effect was significant (*Muscle-Pair F*_(2,22)_ = 5.37, *p* < 0.005). A higher co-contraction (*C*-index) in the TA–SO pair with respect to the RA–TS pair in the Unstable condition was present when the body orientation was 0° [*Muscle-Pair* × *Direction* interaction, *F*_(2,22)_ = 4.03, *p* < 0.005]. Relatively a few significant effects on the *C*-index fail to support Hypothesis 4.

### Comparison of the APA and CPA indices

**Fig. 8:**
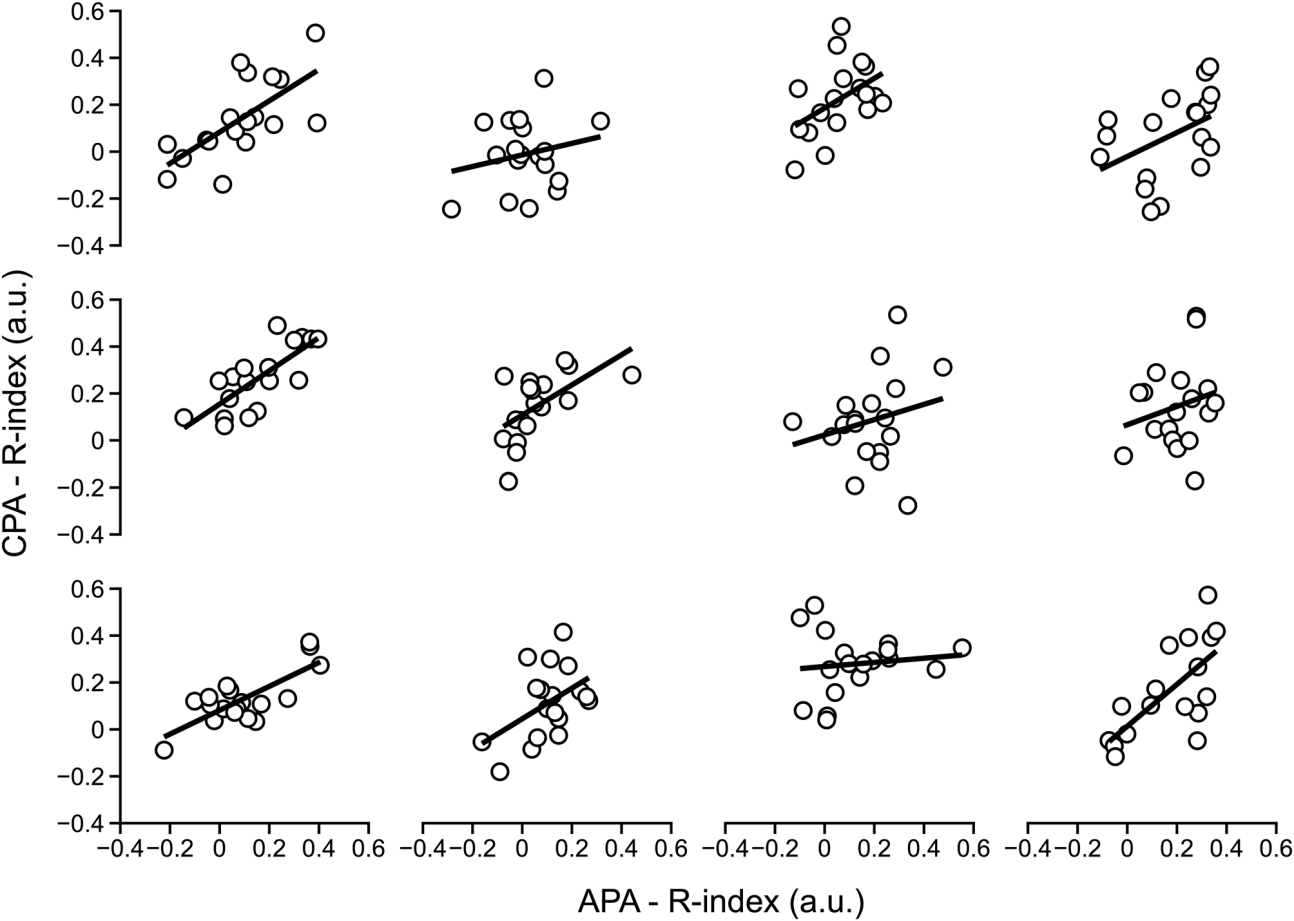
Linear correlations between the indices computed over the anticipatory postural adjustments (APA) and compensatory postural adjustments (CPA) for each subject (12 subjects in total). All regression lines have positive slopes as depicted in each panel. Dots in each panel include the three agonist–antagonist pairs, the two stable–unstable conditions, and the three angles of ball release for a total of 18 points

To test Hypothesis 3, we explored the relationships between the muscle activation indices during the APAs and CPAs (Table 2). Linear regression analysis was performed for each subject separately across conditions, body orientations, and muscle pairs using the values averaged between the two body sides. Examples of the data points, regression lines, and coefficients of determination (*R*^2^) for the *R*-index are presented in Fig. 8. On average, *R*^2^ was 0.467 ± 0.23. The slope of the regression line was positive in all subjects; the Wilcoxson’s signed-rank test confirmed *p* < 0.001. Analysis of the *C*-index produced similar results with a somewhat smaller *R*^2^ (0.312 ± 0.20) and positive slope of the regression line in 10 out of 12 subjects, *p* < 0.01. When the analysis was run using EMG indices for each individual muscle, R^2^ reached even higher values (0.614 ± 0.12), *p* < 0.01. Overall, all the EMG indices showed positively correlated indices for APAs and CPAs.

**Table 2:** *R*-index and *C*-index across the agonist–antagonist muscle pairs

Index	*R*-index		*C*-index	
Muscle-pair	APA	CPA	APA	CPA
TA/SO	0.051 ± 0.012	0.08 ± 0.012	0.152 ± 0.007	0.153 ± 0.008
RF/BF	0.184 ± 0.012	0.161 ± 0.014	0.122 ± 0.005	0.155 ± 0.008
RA/ES	0.125 ± 0.011	0.195 ± 0.016	0.195 ± 0.008	0.208 ± 0.012

Normalized *R*-Index and *C*-Index during both anticipatory postural adjustments (APA) and compensatory postural adjustments (CPA), averaged between the two sides of the body, across conditions and across subjects (mean ± standard deviation)

*TA* tibialis anterior, *SO* soleus, *RF* rectus femoris, *BF* biceps femoris, *RA* rectus abdominis, *ES* erector spinae

**Fig. 9:**
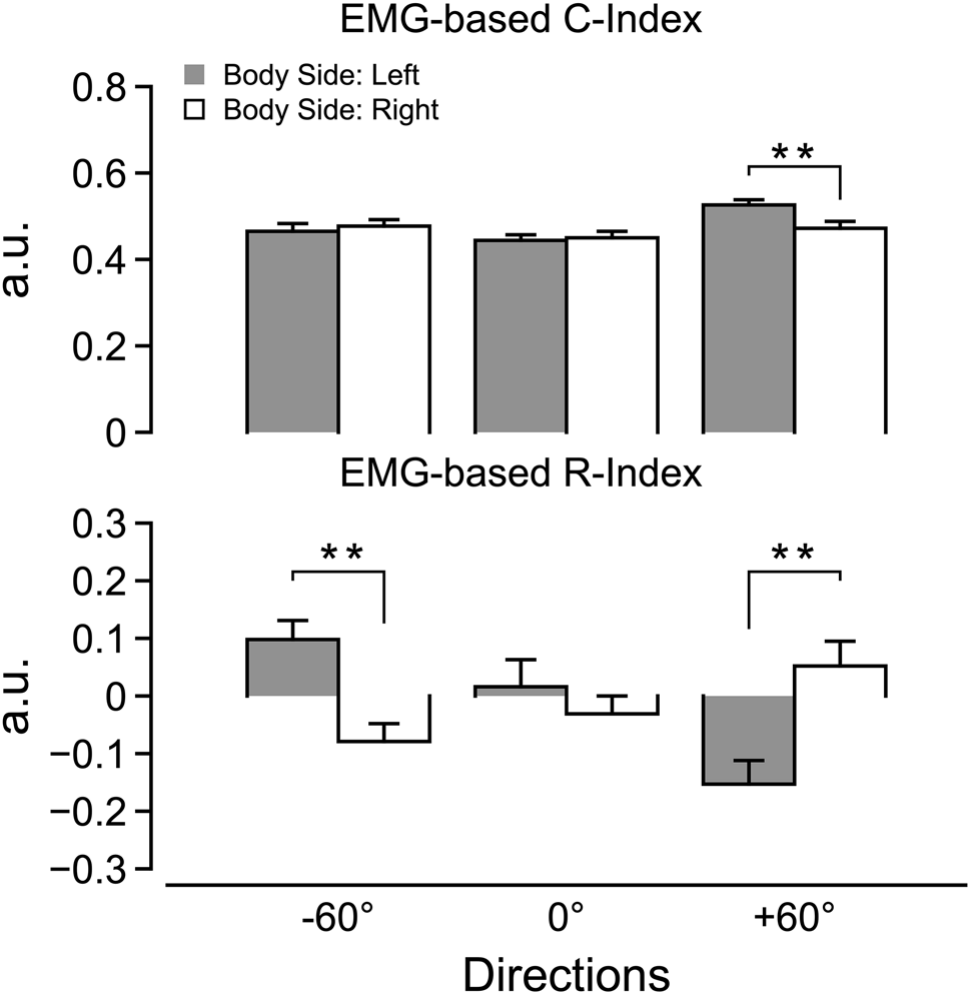
*C*-index (upper row) and *R*-index (bottom row) values related to EMG-based for the agonist–antagonist pairs across all joints (ankle, knee, and hip) and across Stable and Unstable Conditions. Histograms depict the mean value and the standard error. *significant effects at *p* < 0.05, **significant effects at *p* < 0.01

To check whether modulation of baseline EMG could play an important role in the effects on the APA and CPA indices, we performed two extra analyses. First, we explored whether changes in the body configuration and stability conditions had significant effects on the indices computed during the time interval of the baseline, i.e., from –350 ms to –150 ms with respect to the action initiation (see Methods). This analysis revealed a number of significant effects of body rotation and significant interactions. In particular, body rotation led to modulation of the R- and C-indices computed based on the baseline EMG values illustrated in Fig. 9 In particular, there was a significant effect of Muscle-Pair interaction with Direction on R- and C-index (*F*_(4,40)_ = 4.141; *p* < 0.01; *F*_(4,40)_ = 2.723; *p* < 0.05) and on R- and C-indices’ significant interactions for Direction for Body Side (*F*_(2,20)_ = 10.884; *p* < 0.001; (*F*_(2,20)_ = 5.161; *p* < 0.05).

Second, to explore whether these effects could influence our analysis of the EMG-based indices corrected for the baseline EMG levels, we performed analysis of the main indices (R- and C-indices) using EMG indices not corrected for the baseline. All the main effects and interactions described in the previous text were seen in these analyses. We are not presenting them here for the sake of brevity.

## Discussion

Our results provided both expected and unexpected answers to the four specific hypotheses formulated in the Introduction. As predicted by Hypothesis 1, we observed larger adjustments in the indices of muscle activation during both APAs and CPAs in more proximal muscle pairs as compared to the muscle pair acting at the ankle joint. This is not a trivial result, because our perturbation magnitude was modest and it was always 100% predictable–conditions, when the so-called “ankle strategy” may be expected (Horak and Nashner 1986; Diener et al. 1988).

Hypothesis 2 predicted higher indices of muscle activation on the side of the body opposite to the rotation direction. This hypothesis has also been mostly confirmed, although not all the effects reached statistical significance. The contrast between the activation indices on the two body sides was particularly pronounced during the CPAs, but qualitatively similar patterns were seen also during the APAs.

In contrast, Hypotheses-3 and -4 have been falsified by the data. In particular, we saw consistent positive correlations between EMG indices during APAs and CPAs across conditions (cf. Hypothesis 3). This may be seen as unexpected given that negative correlations between similar indices during APAs and CPAs have been reported consistently, although in experiments with manipulations of different task parameters (Krishnan and Aruin 2011; Kaewmanee et al. 2020). Contrary to expectations based on a number of earlier studies (Slijper and Latash 2000, 2004; Piscitelli et al. 2017), we observed statistically significant effects of changes in our task parameters predominantly in the index of reciprocal activation within agonist–antagonist muscle pairs (the *R*-index), not in the index of coactivation (the *C*-index).

Modifications of postural stability did not cause major significant changes in all the indices. It is possible that our Unstable conditions were not sufficiently challenging. However, using smaller support surfaces in the pilot trials made the tasks too challenging for the subjects who could not perform the task consistently without losing balance in conditions that required body rotation.

Overall, our results require reconsideration of some of the established views on the nature and patterns of postural adjustments to self-triggered, predictable perturbations. Reaching consensus on those issues is needed before this new method is used to analyze APAs and CPAs in populations with impaired postural control.

### Control of vertical posture with referent body configurations

Traditionally, studies of the neural control of the vertical posture during standing have been performed, analyzed, and interpreted using mechanical and/or electromyographic (EMG) variables (reviewed in Santos et al. 2010; Krishnan et al. 2011). It has been emphasized, however, since the classical works by Nikolai Bernstein (1947; translation in Latash 2020), that the central nervous system is, in principle, unable to prescribe peripheral mechanics due to the imperfectly predictable body states and external forces, and the length and velocity dependence of muscle forces. Those unpredictable changes in mechanical variables are reflected in equally unpredictable changes in activity of sensory endings sensitive to those variables, which contribute to patterns of muscle activation via reflex loops. As a result, EMG patterns also cannot be seen as reliable indices of central control processes.

Within the theory of the neural control of posture and movement with spatial referent coordinates (RCs) for the effectors (reviewed in Feldman 2015), the neural control is based on specifying time changes in parameters, associated with spatial referent coordinates–parametric control. These parameters can be changed independently of movement mechanics and muscle activation patterns. By its very nature, parametric control of mechanical and EMG variables is indirect, and its effects on those variables emerge in the continuous interaction with the changing external force field, as supported, in particular, by studies of corticospinal effects using transcranial magnetic stimulation (Raptis et al. 2010; Sangani et al. 2011). As of now, continuous measurement of RCs remains a challenge. Experiments with cyclical body actions and reaching movements with reversals have shown global minima of muscle activation levels, which were indicative of RC coinciding with the actual body configuration (Feldman et al. 1998; El-Hage et al. 2023). This method, however, can identify RC coordinates at specific time instants only, not as a continuous control trajectory. As a result, analysis of movement mechanics and muscle EMG indices remains the most common indirect method of assessment of neural control processes. The aforementioned problems with interpretation of EMG indices are partly alleviated when analyzing APAs, which emerge before any measurable consistent changes in mechanics take place (Massion 1992). Still, the spontaneous postural sway makes these indices sensitive to both changes in neural control signals to the muscles and the time-varying contributions of reflex effects. Note that studies of APAs have not been limited to standing but also performed for postural tasks performed by a limb (Struppler et al. 1993) and interpreted within the theory of control with RC (Zhang et al. 2020).

**Fig. 10:**
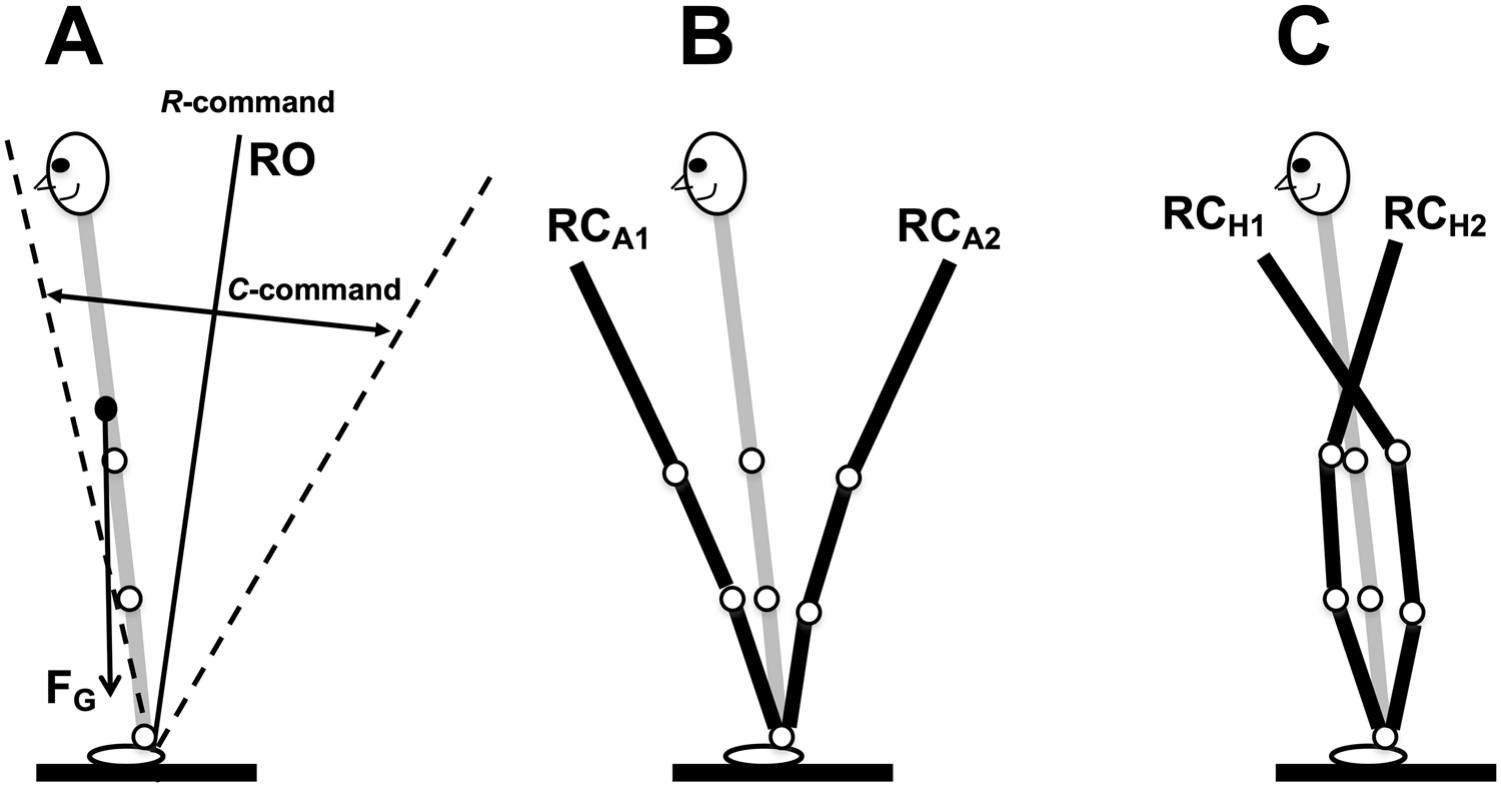
**A** Schematic illustration of the control of vertical posture with the reciprocal and coactivation (R and C) commands. The former defines referent body orientation (RO) in space. The latter defines the range where agonist and antagonist muscles are active simultaneously. At the level of mechanics, it defines apparent stiffness coefficient, which translates the angular units of the difference between RO and actual body orientation (gray image) into moment of force units counteracting effects of gravity. **B** Referent joint configurations (black lines, RC_A1_ and RC_A2_) associated with the “ankle strategy”. **C** Referent joint configurations associated with the “hip strategy” (black lines, RC_H1_ and RC_H2_)

At the highest hierarchical level, the neural control of vertical posture may be viewed as specifying time changes in the *R*-command related to changes in the referent body configuration consisting of both spatial and angular referent coordinates and the *C*-command (see the Introduction and Fig. 1). Figure 10A illustrates referent orientation (RO), produced by changes in the reciprocal command (R-command), and a coactivation command (C-command in Fig. 7A), which defines the spatial zone where both agonist and antagonist muscles are active. We assume in this schematic that rambling does not affect changes in muscle activation within the time intervals of our analysis (150 ms), because it is a relatively slow process with the power primarily limited to frequencies under 0.5 Hz (Zatsiorsky and Duarte 2000). Therefore, the figure assumes that balance during APAs and CPAs is kept with respect to a fixed point. Implementation of this type of control involves a sequence of few-to-many mappings, which lead to the emergence of RCs at the level of individual joints and muscles (Fig. 1B in the Introduction). At the muscle level, RC is equivalent to threshold of the stretch reflex *λ*, as in the classical equilibrium-point hypothesis (Feldman 1966, 1986).

During postural adjustments, changes in the R- and C-commands at the highest hierarchical level can lead to variable mapping on the {R; C} pairs at the joint level in a task-specific manner. Variability in this mapping has been reflected in the composition of muscle groups identified with various matrix factorization techniques (addressed as “muscle synergies” or “muscle modes”, reviewed in Ivanenko et al. 2006; Ting and McKay 2007; Lacquaniti et al. 2012; Latash 2021). While the composition of such muscle modes remains robust within a range of task changes, both the number of such modes and their composition may change with such factors as task stability (Danna-dos-Santos et al. 2008) and extensive practice (Asaka et al. 2008). There are major changes in the muscle mode composition with tasks that require quick shifts in the COP coordinate and those that require a quick pulse of shear force in the same direction (Robert et al. 2008). The former are associated with joint referent configuration shifts resembling the patterns addressed as the “ankle strategy” (Fig. 7B), while the latter suggest changes in the joint referent configuration resembling the “hip strategy” (Fig. 7C) (cf. Horak and Nashner 1986).

### Interactions between the reciprocal and coactivation commands

The two basic commands, R and C, are both important in their effects on mechanical and EMG patterns. A hierarchical relation between the two commands was suggested with the R-command being hierarchically higher (Levin and Dimov 1997). For example, imagine that a person generates a certain level of voluntary muscle coactivation at a static position of a joint, i.e., a non-zero value of the C-command. If this person performs a quick joint movement to a new position with a change in the R-command, the C-command will be transferred to the new spatial position of the joint. On the other hand, recent studies of motor unit recruitment patterns during cyclical force production in isometric conditions have suggested that the C-command can show consistent modulation within the force cycle accompanied by relatively irregular, subject-specific changes in the R-command (Madarshahian and Latash 2022).

Typical patterns of muscle activation during APAs and CPAs suggest that their neural control involves complex patterns of RCs at the level of the major joints along the body vertical axis involving changes in both *R*- and *C*-commands. For example, during APAs, some of the agonist–antagonist muscle pairs show reciprocal patterns of muscle activation, while other muscle pairs show simultaneous EMG bursts expected from a quick change in the *C*-command. EMG patterns in agonist–antagonist muscle pairs during both APAs and CPAs switch from more common reciprocal patterns to predominantly coactivation patterns in challenging tasks and also in populations with impaired postural control (Slijper et al. 2002; Slijper and Latash 2004; Valle et al. 2013; Lee et al. 2015).

Studies of adjustments in characteristics of APAs to task modifications have emphasized modulation of an EMG-based index related to the *C*-command (Slijper and Latash 2000, 2004). Moreover, in conditions of uncertainty, for example when the direction of a self-triggered perturbation is not known in advance, this index increases at the expense of the index reflecting changed in the *R*-command (Piscitelli et al. 2017). Based on those results, we expected the *C*-index in our study during the APAs to show modulation with task changes. This was not the case, however. More statistically significant effects were seen in the analysis of the *R*-index. Note that both indices reflect two factors: the *R-* and *C*-commands at the task level and mapping of those commands to the {*R*; *C*} pairs at the joint level (Fig. 1B). It is possible that, depending on specific features of the task being manipulated, the central nervous system adjusts primarily the *C*-command or the *R*-command to fit the task demands and/or that the pattern of mapping of the task-level commands to those that define muscle activations changes. Although body rotation led to significant effects on the baseline EMG indices (see Fig. 9 in Results), our analysis of the indices corrected and not corrected for the baseline produced statistically similar results. Therefore, we feel safe to conclude that the described changes in the EMG-based indices during the APA and CPA intervals are not due to the modulation of the baseline EMG.

For example, uncertainty on the perturbation direction makes using large *R*-command shifts dangerous, because their effects can sum up with the effects of the perturbation if the perturbation direction happened to be guessed wrongly (Bertucco et al. 2021; Nardello et al. 2021; Piscitelli et al. 2017). Increasing the *C*-command during APAs is more universal, because adjusting this command does not produce large net changes in the resultant moment of force but, instead, modifies the apparent stiffness of the effector thus reducing its spatial deviations independently of the perturbation direction. In contrast, if modifications of the perturbation characteristics are known in advance, as it was the case in our study, adjustments of the *R*-command lead to efficient changes in the resultant moment of force as required by the new conditions.

Overall, the interactions between the *R*- and *C*-commands represent a complex group of phenomena, which cannot be reduced to a universal hierarchical relation over tasks and conditions. During movements in isotonic conditions, changes in the *R*-command may be hierarchically higher. However, during accurate force production in isometric conditions and during whole-body tasks performed while standing, the importance of adjustments in the two basic commands may change in a task-specific manner.

### Feed-forward and feedback postural control

Within the theory of control with RCs, any action begins essentially in a feed-forward way, because results of the issued neural commands become available at a time delay. In a healthy person, implementation of control variables, i.e., their conversion into movement mechanics, always involves feedback loops from peripheral sensory endings. In studies of posture, the term feed-forward control has been used traditionally to address the neural processes leading to the generation of postural adjustments to future events, such as postural perturbations, expected based on the actor’s experience and available information. Since the ability to predict is never perfect, in particular given the time-varying state of the body (postural sway), APAs are never able to compensate for the expected effects of the predicted perturbations perfectly. Available information suggests that, typically, the central nervous system facilitates smaller APAs, such that there is always residual perturbation acting in the predicted direction (reviewed in Massion 1992). Besides, under some conditions, APAs may themselves turn into threatening factors, e.g., when the actor stands on a surface with narrow support (Slijper et al. 2002).

The residual perturbation of vertical posture is handled by CPAs, reflections of pre-programmed changes in the joint referent configuration leading to bursts of activation in postural muscles, which emerge at time delays shorter than the simple reaction time (Kaewmanee et al. 2020; Kaewmanee and Aruin 2022). A number of studies have provided evidence for negative covariation between the magnitudes of APAs and CPAs (Krishnan and Aruin 2011; Kaewmanee et al. 2020). This may be seen as an expected result given that CPAs have to handle residual perturbations attenuated by APAs. In our study, the predominant relation between the APA and CPA magnitudes was positive, i.e., conditions with stronger APAs were associated with stronger CPAs. This may be interpreted as a consequence of the relatively large variation in the effective perturbation in the anterior–posterior direction across conditions. Indeed, although the load magnitude was kept constant, body rotation away from 0° produced smaller effective perturbations in the sagittal plane. This could result in both smaller APAs (since the effects of body rotation were experienced by the subjects) and smaller residual perturbations leading to smaller CPAs. Note that positive covariation between the EMG indices computed for APAs and CPAs were observed in each of the subjects, suggesting that inter-subject variability in those indices was not a defining factor.

Overall, the effects of torso rotation on postural adjustments to a standard perturbation triggered by a standard action differ from what could be expected based on earlier studies. In particular, these effects lead to significant modulation of the *R*-index and positive covariation between the EMG indices of feed-forward and feedback adjustments, APAs and CPAs. Based on a number of studies of the effects of neurological disorders, in particular those associated with subcortical dysfunction, on APAs and CPAs (e.g., Bazalgette et al. 1986; Bouisset and Zattara 1990; Horak and Diener 1994), we expect the described characteristics to be sensitive to a range of conditions associated with postural instability—a topic for future studies. It is possible that some of the effects found in our study were due to changes in vestibular signals associated with head rotation. Note that effects of vestibular signals on referent body configuration have been reported (Zhang et al. 2018), and these effects could contribute to the redistribution of muscle activation in our experiments.

## Data Availability

Data will be available on reasonable request.
